# SiO_2_ thin film growth through a pure atomic layer deposition technique at room temperature†

**DOI:** 10.1039/d0ra01602k

**Published:** 2020-05-11

**Authors:** D. Arl, V. Rogé, N. Adjeroud, B. R. Pistillo, M. Sarr, N. Bahlawane, D. Lenoble

**Affiliations:** Luxembourg Institute of Science and Technology 41 rue du Brill L-4422 Luxembourg didier.arl@list.lu

## Abstract

In this study, less contaminated and porous SiO_2_ films were grown *via* ALD at room temperature. In addition to the well-known catalytic effect of ammonia, the self-limitation of the reaction was demonstrated by tuning the exposure of SiCl_4_, NH_3_ and H_2_O. This pure ALD approach generated porous oxide layers with very low chloride contamination in films. This optimized RT-ALD process could be applied to a wide range of substrates that need to be 3D-coated, similar to mesoporous structured membranes.

## Abstract 2

In this study, less contaminated and porous SiO_2_ films were grown *via* ALD at room temperature.

## Graphical abstract

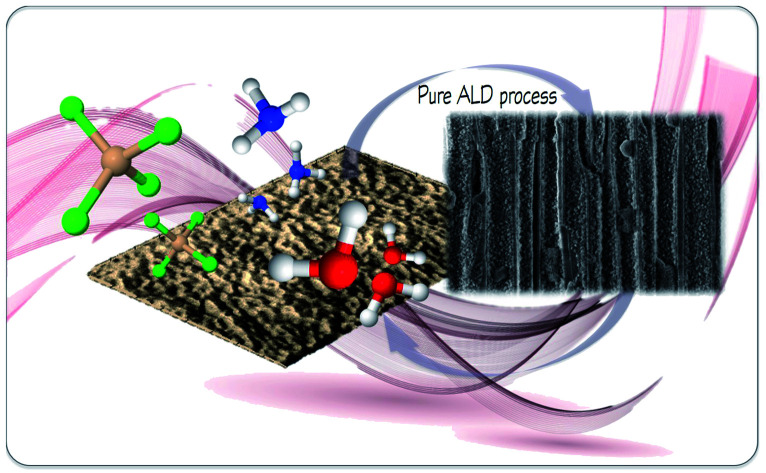

## Introduction

Silicon dioxide (SiO_2_) and more generally ultra-thin oxide films have been extensively described as good components for modern nanotechnologies such as dielectric materials in silicon microelectronic devices,^1,2^ anticorrosion films^3^ or non-exhaustive applications of nanoscale films in catalysis. The environment- and human-friendly nature of SiO_2_ induces its wide use in protective layers for antisticking, antifogging, self-cleaning or water repellency.^4–7^ Various techniques such as chemical vapor deposition,^8^ lithographic patterning,^9^ electrochemical deposition^10^ or sol–gel^11,12^ were investigated to prepare superhydrophobic SiO_2_ by tuning surface roughness or energy. SiO_2_ is consistently known for its application in protective or gate insulator coatings^13^ and interfacing high-*k* (ref. 14–19) or surface passivation materials.^20–23^ The increased demand for transparent active materials at the nanoscale justify the need for a deposition technique compatible with sensitive pre-deposited underlying layers, flexible plastic devices or high aspect ratio substrates.^24–28^ Therefore, atomic layer deposition (ALD) is considered as one of the most suitable techniques for its performance in terms of sub-nanometer thickness control and penetration coating into deep trenches or mesoporous structures.

SiO_2_ thin films obtained through ALD have widely been described as binary surface reactions dealing with various types of precursors Si(C_2_H_5_O)_4_,^29^ SiCl_4_ (ref. 13 and 30–33) or Si(NCO)_4_ (ref. 34) with H_2_O; CH_3_OSi(NCO)_3_ (ref. 35 and 36) with H_2_O_2_; Si(NCH_3_)_2_)_4_, ((CH_3_)_2_N)_3_SiH^37^ or SiH_2_(NEt_2_)_2_ (ref. 38)). Besides the necessity to work at high temperatures (*i.e.* >100–350 °C), most of the reactions require large reactant exposure of ≥10^9^ L (1 L = 10^−6^ Torr s) with a growth rate of^59^ ∼1–2 Å per cycle.^39^ One approach to decrease the deposition temperature and the level of contamination is to use plasma-enhanced-ALD (PE-ALD).^40,58,59^ It is possible to decrease the temperature below 50 °C, and these SiO_2_ films are described as excellent candidates for thin film encapsulation in organic devices or TFT gate insulators due to the absence of impurities and good electrical properties.^41–43,60^ Currently, the use of amino ligands as precursors leads to promising results, even on large surfaces; however, a final annealing step at 900–1000 °C is required to decrease interface defects or carbon contamination.^44,45^ Many efforts have been done, in terms of parameters and choice of precursors, in order to optimise ALD process, for the growth of SiO_2_ coatings at high temperatures. Nevertheless, it is commonly agreed that there is a strong interest in the development of the process at room temperature. George *et al.* described the atomic layer-controlled growth using SiCl_4_ and H_2_O many times.^13,31,44,46^ They demonstrated that a reaction catalyzed using Lewis bases such as pyridine (C_5_H_5_N) or ammonia (NH_3_) avoids large precursor flow rates and can only occur close to room temperature. Nevertheless, in these studies, C_5_H_5_N or NH_3_ were never really considered as “precursors”. The proposed mechanism, which took into account the hydrogen bonding between the Lewis base and either the SiOH* surface species or the H_2_O reactant, was studied by considering the global residual pressure of the continuous flow of the catalyst. Moreover, the secondary reaction of the catalyst reservoir (continuous flow), available in the reactor with HCl as the byproduct, drastically increased the probability of the inclusion of contaminants in the film. Therefore, a sequential approach could enhance the quality of the film and the understanding of the role of the catalyst.

This paper describes a pure ALD study of SiO_2_ using the optimized sequential exposure of SiCl_4_, H_2_O and NH_3(g)_ precursors at room temperature. The ALD mode was confirmed by tuning the exposition of each precursor and the related purges. The initiation of the exposition was followed by using the residual gas analysis (RGA) mass spectrometer. Atomic growth control was investigated by the *in situ* Quartz Crystal Microbalance (QCM) and X-ray Photoelectron Spectroscopy (XPS), Dynamic-Secondary Mass Ion Spectroscopy (D-SIMS) and X-ray Diffraction (XRD) post-characterizations. A comparison between our investigation and the state-of-the-art of low temperature ALD SiO_2_ synthesis revealed the possibility to deposit ultra-thin films with very low contaminations at room temperature. The film conformality is shown and the capability of this optimized binary reaction, to be used on various types of temperature-sensitive supports with high aspect ratios, is confirmed.

## Materials and methods

ALD processes were carried out in a TFS200-Beneq reactor in the planar configuration at a base pressure of 0.3 mbar. SiO_2_ thin films were then deposited on Si substrates, preliminary prepared by a standardized cleaning procedure established by Radio Corporation of America (RCA). The deposition reactor was equipped with a QCM (Neyco) for the gravimetric monitoring of the film growth. The QCM was fixed to the central part of the substrate holder. A quadrupole mass spectrometer, Vision-2000C, MKS-instrument, was assembled at the outlet of the deposition reactor to monitor the exhaust gas composition. SiO_2_ thin films were obtained at room temperature using SiCl_4_ and H_2_O as precursors. The vaporized precursors were transferred to the ALD reaction chamber with N_2_ as the carrier gas. SiCl_4_ was purchased from Sigma Aldrich and used as-received. Both canisters containing the precursors were maintained at 19 °C during deposition. NH_3_ gas (<99.9%), used as a catalyst, was injected into the reactor under 1 bar pressure.

The morphology and thickness of the obtained samples were characterized using a FEI Heliosnanolab 650 Focused Ion Beam Secondary Electron Microscope (FIB-SEM). The structure of the films deposited on dedicated Kapton tape was probed by small-angle X-ray scattering (SAXS) using an X-ray Diffractometer (X'Pert Pro (Panalytical)) equipped with a focusing mirror and a Pixcel 1D detector in the transmission mode. The elemental composition depth profile was assessed using D-SIMS (Cameca, IMSLAM); however, the quantification was performed by XPS (Thermo VG Scientific, MicroLab 350) using Al Kα source.

## Results and discussion

### Catalytic SiO_2_ RT-ALD growth

SiO_2_ films obtained at room temperature have already been prepared by the sequential exposure (ABAB…) of two reactants (A and B). Many well-known precursors require high deposition temperatures, plasma or highly reactive co-reactants such as ozone gas.^47^ Despite a low enthalpy of reaction, SiCl_4_ usually reacts with water (oxidant species) at high temperatures (>325 °C).^13^ The comparison of thermal ALD and room temperature processes reveals a higher growth rate/ALD cycle in favour of room temperature reactions (∼2 Å per cycle) (Fig. S1, ESI†).

The amount of contaminants integrated in RT-SiO_2_ films is inherently dependent on the way of tuning the surface exposure to precursors. Based on studies by George *et al.*,^31^ we investigated the growth of SiO_2_ at room temperature (RT)-ALD by alternatively exposing the surface to SiCl_4_ and H_2_O under a constant flow of NH_3_. The exposure time was fixed at 90 s for SiCl_4_ with a purge time of 1 min. H_2_O exposure was fixed at 90 s with a purge time of 5 min to ensure a perfect saturation of the surface.^31^ The *in situ* monitoring of the film growth obtained by the QCM is shown in Fig. S2, ESI.† As already described,^31^ the reduction reaction of SiCl_4_ with water is depicted through the minimum of SiCl_4_ weight gain (Fig. S2b, ESI†) and the longer reaction of the NH_3_–H_2_O mixture (Fig. S2c–e, ESI†). Then, as shown in Fig. S2b, ESI,† the gain of mass that is observed in one ALD cycle is predominantly obtained from the half reaction of H_2_O. Nevertheless, the growth rate of ∼1.5 Å per cycle (297 nm/2000 cycles), experimentally obtained through this process with a constant flow of NH_3_, tends to reach the 2 Å per cycle value described by George *et al.*^31^

**Fig. 1:**
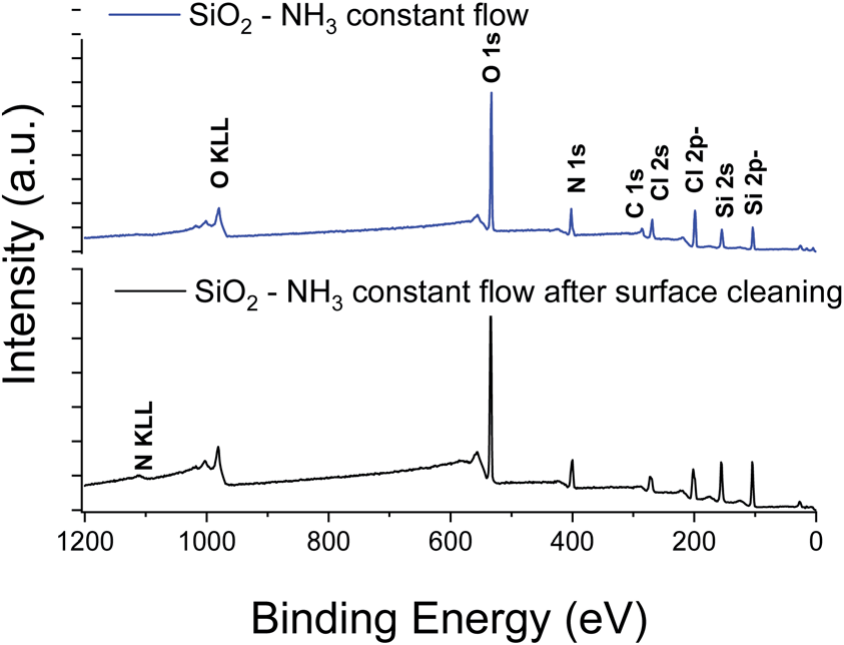
XPS survey spectra of SiO_2_ thin films obtained with a constant flow of NH_3_.

XPS elemental analysis of the SiO_2_ film (deposited on Al_2_O_3_/Si) (Fig. 1) shows the presence of carbon, nitrogen and chlorine in addition to silicon and oxygen within the SiO_2_ deposited film. A depth profiling analysis reveals that the carbon is restricted to the surface of the film.

The asymmetry of the C 1s peak suggests the presence of C–H, C–O–C or C=O bonds at the surface due to air exposure. The Si 2p peak of the Si–O layer appeared at 104.4 eV with a 1.77 eV (broadened up to 2.22 eV due to charge effects during the depth profiling) full width at half maximum (FWHM). The sharp and symmetric Si 2p peak centred at ∼104 eV suggests an oxidation state of +1, which was attributed to the presence of SiO_2_. A high contribution of the byproducts of the reaction is detected through the presence of Cl and N elements in the films. The Si/Cl ratio increased from ∼1.3 at the surface to 3.2 in the bulk of the film (Table 1).

**Table 1:** XPS quantification of elements present in the SiO_2_ thin film obtained with a constant flow of NH_3_

Name	At%	At% (depth profiling)
Si 2p	17.6	29.5
O 1s	45.9	47.7
N 1s	16.5	13.5
Cl 2p	13.6	9.1
C 1s	6.4	<1

**Fig. 2:**
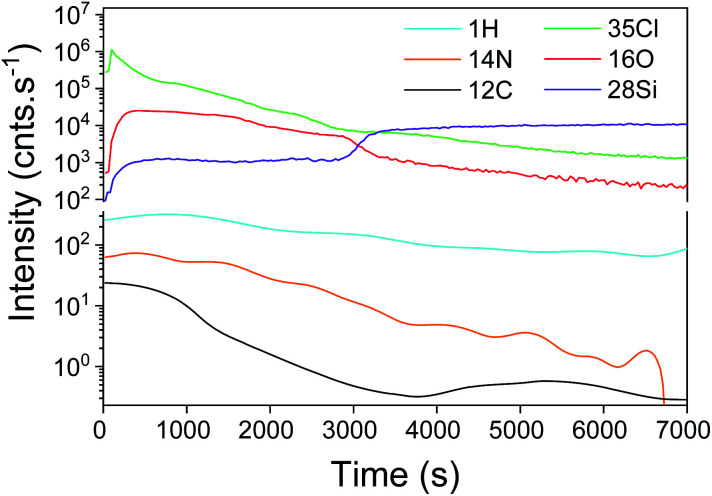
SIMS depth profile of the SiO_2_ film obtained with a constant flow of NH_3_. A thickness of 297 nm is calculated by the sputtering time.

Furthermore, the reaction of the lone pair of active –Cl with the hydrogen of water induced a significant formation of HCl. The higher detection limit of D-SIMS was used to screen the chemical elements present in the “bulk” of the film, particularly for light elements such as hydrogen. As shown in Fig. 2, all typical elements of the deposited film, *i.e.* Si, O, Cl, N, H and C, are detected. The intensity of Cl is similar to that of Si and O while a difference in the intensity is observed for H, N and C. It can be seen from the depth profile analysis that the signal of C decreases rapidly, which is in agreement with the XPS results. As H% is roughly constant, the slow decrease in N and Cl tends to confirm that a part of the film is composed of NH_4_Cl contaminants.

### Contaminant inclusion mechanism

George *et al.* described the mechanism of a catalysed binary reaction that spontaneously takes place in the presence of pyridine or NH_3_ as a Lewis base agent.^30,48^ The hydrogen bonding between the Lewis base and SiOH* (surface species) or H_2_O allows the reaction to be performed at room temperature (Fig. S3, ESI†). Compared to high temperature processes that use large precursor exposures (>10^3^ Torr s), SiO_2_ RT-ALD takes place owing to the strong nucleophilic attack of the oxygen from (i) SiO̲H* on SiCl_4_ and that from (ii) H_2_O on SiCl*.^46^ Nevertheless, to the best of our knowledge, no specific data have been reported on the variation of the chemical composition and the morphology of such films. Based on the catalytic effect of NH_3_, it can be clearly deduced that a constant flow of NH_3_ statistically ensures a maximized reaction of –O on all –O–Si–(Cl)_*n*_ available sites. Nevertheless, the perfect delimitation of the exposure windows at room temperature could be enhanced by working in a non-conventional high vacuum state (<10^−6^ Torr). As it is not the case for standard ALD reactors, we attempted to understand and control the contaminant inclusion mechanism in the pulsed NH_3_ regime. Thus, the state-of-the-art production of SiO_2_ at RT using a constant flow of NH_3_ has been compared to pulse NH_3_-catalysed RT-ALD. Inspired by the reactivity of chlorinated precursors described by Damyanov *et al.*,^49^ the amount of contamination could be cautiously explained by the functionality *x* of the adsorbed species at the surface explained hereafter:*x*(≡Si–OH) + SiCl_4_ → (≡Si–O)_*x*_SiCl_4−*x*_ + *x*HCl*x* = 1: monofunctional, *x* = 2: bifunctional and *x* = 3: trifunctional.

The injected precursor SiCl_4_ reacted with the surface (≡) hydroxyl species. Moreover, the competition between the single bond case (*x* = 1) and multiple bonds (1 < *x* ≤ 3) was directly linked to the stagnancy of precursors in the ALD regime. As far as the concentration of hydroxyl groups on the surface increased, the saturation of H_2_O directly enhanced the formation of HCl. Along with the constant flow of NH_3_, the ∼2.2 Si/N ratio measured by XPS in the bulk of the film indicates a strong nitrogen contamination exceeding acceptable limits, especially through the inclusion of NH_4_Cl salt. As indicated by George *et al.*,^31^ this salt is formed as a result of the NH_3_ catalyst complexing with HCl. Because of the vapour pressure of the NH_4_Cl salt (*i.e.* 4 × 10^−5^ Torr (ref. 50)), some quantity of the salt remained within the film. In that context, note that compared to an inert gas, using NH_3_ as a carrier gas may not contribute to a pure ALD process performed at RT. Indeed, a significant contamination of the surface is attributed to the excessive dose of NH_3_. The contamination depicted here confirms the already described importance of adjusting the quantity of NH_3_ to limit the reaction with HCl.^31^ Thus, we considered that pulsing NH_3_ similar to the other precursors could minimize unfavourable reactions at room temperature.

### Low contamination SiO_2_ growth under pulsed NH_3_

#### Dense oxide under pure ALD regime

**Fig. 3:**
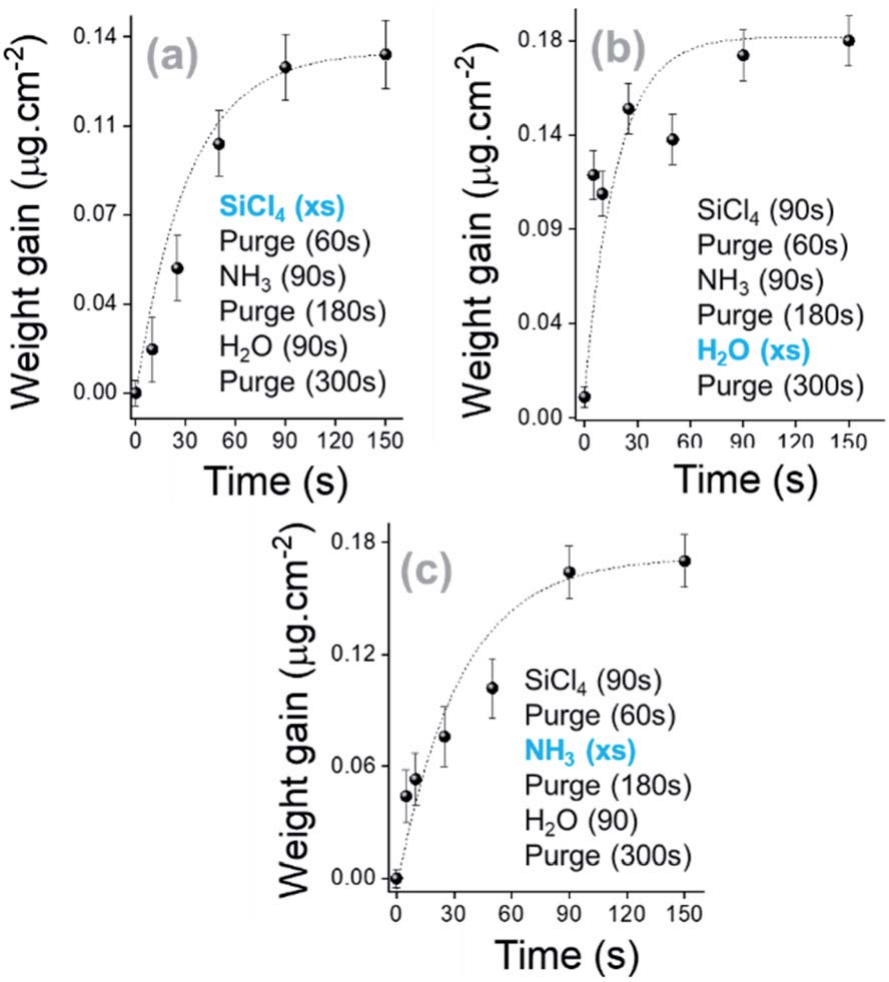
Saturation curves of SiCl_4_, H_2_O and NH_3_ along the SiO_2_ thin film growth. The kinetic growth is shown for 5 cycles with sequential exposure of the surface.

Based on the same chemistry used in the previous part, each chemical involved in the following process has been considered as a precursor. This indicates that an adequate separation of each pulsed chemical has been guaranteed. The purge of the reactor has been optimized using the appropriate ratio of carrier gas flow/reactor base pressure (<2 Torr). Any overlap between each precursor pulse has been prevented by checking the injection with the integrated RGA. Fig. 3 shows the ALD saturation curves at RT for SiCl_4_ (a), H_2_O (b) and NH_3_ (c) precursors. According to the diagrams, the saturation of all precursors occurs after exposure for 90 s. The N_2_ purging time between SiCl_4_ and NH_3_ precursors has been fixed at 90 s. The appropriate purging time after water exposure was then determined by RGA (H_2_O: *m*/*z* = 18 uma) analysis using a systematic variation process (Fig. S4, ESI†). After 300 s purging time, water was completely removed from the reactor.

**Fig. 4:**
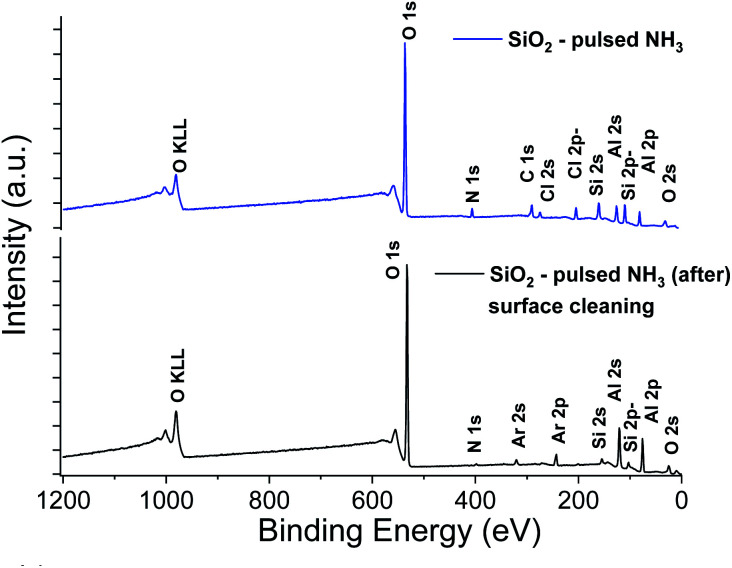
XPS survey spectra of SiO_2_ thin film obtained with a sequential 90 s exposure of SiCl_4_, NH_3_ and H_2_O precursors. Upper panel corresponds to the signal of the raw film and lower panel to the film after surface cleaning.

Based on the trends observed in Fig. 4, the growth of a SiO_2_ film in a pure ALD regime at RT has been investigated. SiCl_4_, NH_3_ and H_2_O exposure times were fixed at 90 s and extended purges were applied after NH_3_ and H_2_O pulses at 180 s and 300 s, respectively. As shown in Fig. S5a, ESI,† the 0.02 μg cm^−2^ per cycle weight gain is 30 times lower than the process with a constant flow of NH_3_ (Fig. S2, ESI†). Nevertheless, the injection of NH_3_ and H_2_O precursors significantly contributes to a certain gain of mass (Fig. S5 panel b, ESI†), and then a growth rate of 0.5 Å per cycle is obtained for 500 deposition cycles. It can be observed that the high H_2_O mass adsorbed during the interaction of H_2_O molecules with active complexes at the surface ends through the efficient replacement of chlorine by hydroxyl groups (Fig. S3, ESI†).

The XPS elemental analysis (Fig. 4) still shows the presence of chlorine, nitrogen and carbon in addition to silicon and oxygen. The amount of contaminants (Table 2) is nonetheless substantially decreased. Firstly, the Si/Cl ratio increased from ∼4 (surface) to ∼8.7 in the bulk of the film. Secondly, compared to the films obtained with a constant flow of ammonia, the Si/Cl ratio improved significantly (threefold). Moreover, the Si/N ratio increased from 1.1 to 3.8 (2.2 to 5.9 inside the film). This indicates a limited reaction between HCl and NH_3_ to form NH_4_Cl. The best fitting procedure of the high-resolution spectrum of N 1s reveals a single binding energy peak at 401.1 ± 0.3 eV, corresponding to NH_3_^+^. This confirms the formation of the NH_4_Cl salt, and the small amount of detected Al is attributed to the alumina sub-layer (*i.e.* SiO_2_/Al_2_O_3_/Si).

**Table 2:** XPS quantification of elements present in the SiO_2_ thin film obtained with a pure ALD regime

Name	At%	At% (depth profiling)
Si 2p	12.9	4.1
O 1s	54.2	59.7
N 1s	3.6	0.7
Cl 2p	3.2	0.5
C 1s	11.4	<1.0
Al 2p	14.7	34.9

**Fig. 5:**
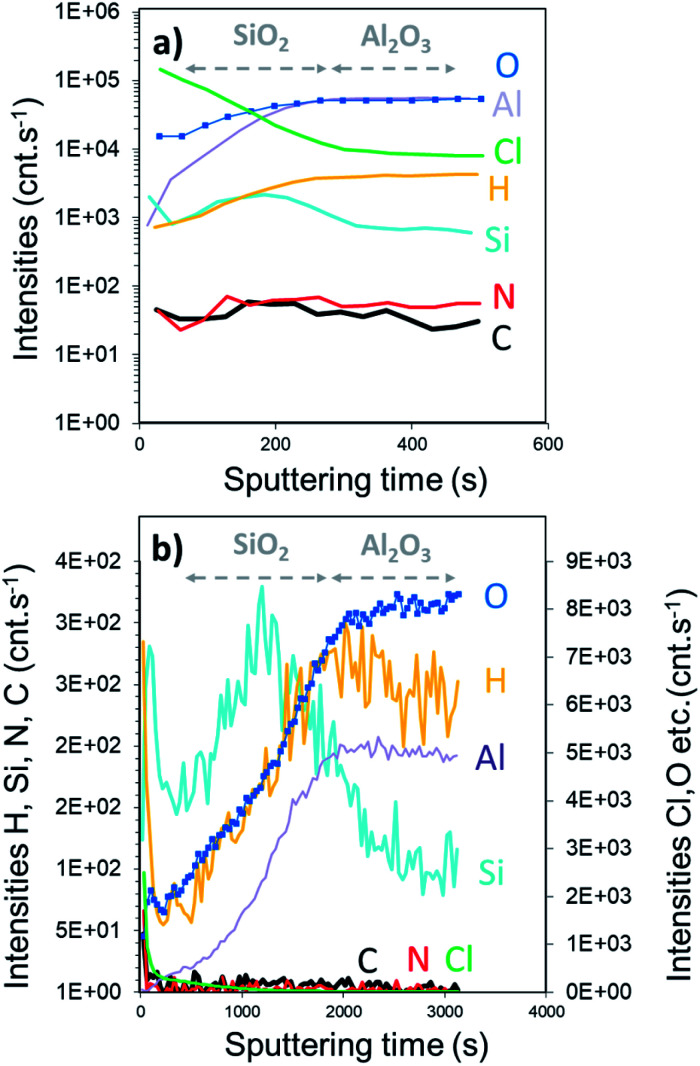
SIMS depth profile of pure ALD SiO_2_ film obtained with 90 s pulse of SiCl_4_; NH_3_ and H_2_O precursors and extended N_2_ purges for 60, 180 and 300 s. Fast and slow sputtering rate are shown in panels (a) and (b), respectively.

The SIMS depth profile of the SiO_2_ film is shown in Fig. 5. The intensity of chlorine decreases ∼30 times faster than the process performed with the constant NH_3_ flow. In fact, less than 100 s sputtering is needed to decrease the intensity below 1 × 10^5^ cnts per s compared to ∼2800 s for the NH_3_ constant flow process. Moreover, the intensity of nitrogen seems to be in the same range of 10–100 cnts per s. Compared to the XPS results, this corroborates the formation of the NH_4_Cl salt. Note that the intensity of Si is higher than that of Al, confirming the coating process of SiO_2_ on Al_2_O_3_. From the depth profile, we can estimate the SiO_2_ film thickness to be around 25 nm. Based on the XPS and SIMS results, it can be assumed that this RT process is optimized in terms of surface exposure. Nevertheless, the residual traces of HCl still react with NH_3_ because of the difficulties to purge H_2_O or NH_3_ at RT. Moreover, small quantities of byproducts, such as NH_4_Cl, were consequently integrated into the film.

**Fig. 6:**
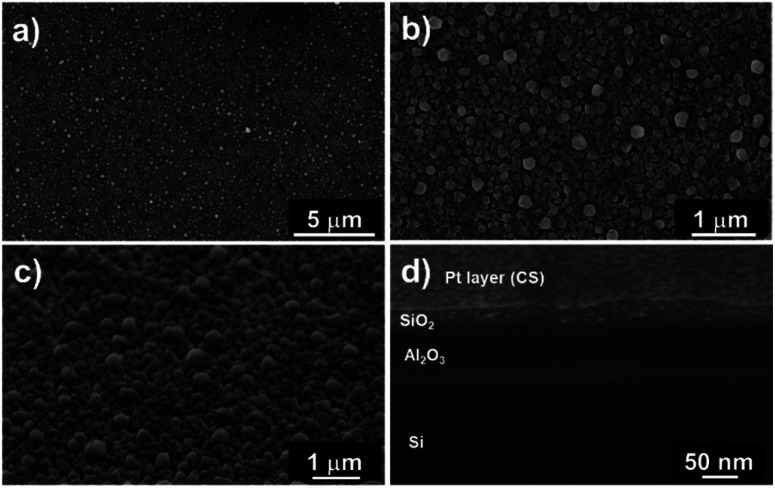
SEM images of the pure ALD SiO_2_ film processed for 500 cycles at different magnifications: (a and b) top and (c) 45° tilted view of the entire oxide film. (d) FIB cross-section images reveal a dense state of the SiO_2_ film with an inhomogeneous crystallisation due to the inclusion of contaminants and the low growth rate of 0.5 Å per cycle.

Fig. 6a and b show top-view SEM images of the SiO_2_ film. We observed a rough layer with grain sizes of up to 200 nm. This roughness is highlighted in the 45° tilted view (Fig. 6c).

Furthermore, cross-section analyses evidence the presence of a compact film (Fig. 6d). From these pictures, we can conclude that this pulsed NH_3_ growth process leads to dense but rough SiO_2_ thin films. A thickness of 30 ± 5 nm is measured through the cross-section, close to the 25 nm value deduced from the SIMS analysis. This leads to a lower growth rate of ∼0.5 Å per cycle related to the lower weight gain observed with QCM (*i.e.* 30× lower than the SiO_2_ film processed under a constant flow of NH_3_). Nevertheless, the irregular surface aspect reveals that the process does not correspond to a pure ALD growth mode, as expected.^51–53^ This peculiar non-homogeneous growth at RT suggests that the surface reaction is in competition with the integration of contaminants. The self-limiting process actually promotes the deposition of species onto the substrate and onto the deposits (*e.g.* islands) with equal probability.^54–56^ The inclusion of contaminants at a sub-atomic growth rate (*i.e.* <1 Å per cycle) could explain the morphology of the obtained film. Moreover, the high amount of –OH surface groups could affect the dehydroxylation/rehydroxylation equilibrium (section: Contaminant inclusion mechanism), leading to the production of a higher quantity of HCl in the case of trifunctional bonds. Nevertheless, the oxide thin film displays a significant density in volume with limited inhomogeneity. This is in line with the sub-atomic growth rate mechanism surrounded by limited contamination. The tailoring of ALD parameters in this RT-SiO_2_ growth process shows a substantial adaptability in terms of morphology and chemical composition. These results suggest that a tuning of the growth parameters could influence the crystallisation. Hence, different types of SiO_2_ layers could be processed at RT.

#### Porous oxide under limited ALD regime

As described in the previous section, less contaminated SiO_2_ can be produced by adjusting the surface exposure of SiCl_4_, NH_3_ and H_2_O precursors. Furthermore, the effect of limited exposure on the composition and the morphology of the film has been investigated. Hence, the process has been tuned to maintain low level of contaminants in an ALD non-saturation regime. The precursor exposure has been decreased to a minimum value for SiCl_4_ (*i.e.* 100 ms) in agreement with a low contamination strategy. Then, according to RGA results, the exposure time of NH_3_ and H_2_O was fixed to 2 s for both with a purge of only 10 s using 300 sccm of N_2_.

As shown in Fig. S6, ESI,† a growth rate of 1.54 μg cm^−2^ per cycle was obtained. Compared to the process described in the previous section, the exposure reaction used here generates ∼50 times higher weight gain. In order to disentangle any physico-chemical influence from the substrate, silicon oxide films were grown on a pre-characterized barrier layer. Hence, SiO_2_ growth was investigated on two different sub-layers, *i.e.* TiO_2_ deposited by ALD and Si bulk.

**Fig. 7:**
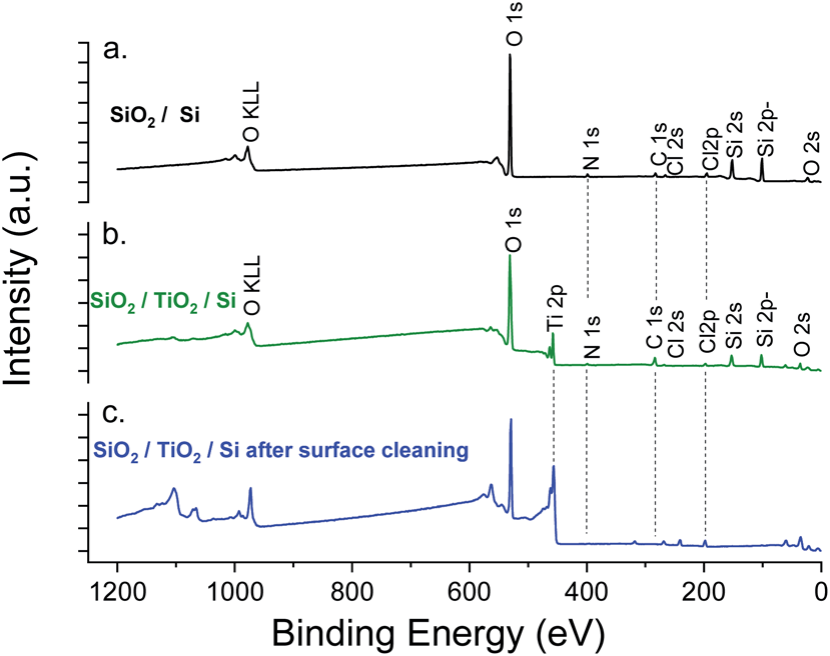
XPS survey spectra of SiO_2_ thin film obtained with a sequential exposure of SiCl_4_, NH_3_ and H_2_O precursors. SiO_2_ film deposited on (a) Si wafer, (b, c) TiO_2_/Si (80 nm) before and after surface cleaning respectively.

Fig. 7 shows the XPS experiment results. As expected, Cl, C, N elements were detected in both samples. For SiO_2_ deposited on TiO_2_, the detection of Ti 2p before etching confirms the low thickness of the film.

However, the percentage of chlorine was clearly maintained below the limit of 3% (obtained for the previous process with extended exposures) (Table 3). The higher chlorine concentration observed after etching (3.79 at%) was attributed to the chlorine inherent to the TiO_2_ ALD process. This was confirmed by the low chlorine concentration for SiO_2_ deposited directly on the silicon wafer (Table 3, SiO_2_/Si). In order to screen the composition and morphology of the film, thicker SiO_2_ layer (2500 cycles) were processed on a chlorine-free material, *i.e.* Al_2_O_3_ (50 nm) on Si.

**Table 3:** XPS quantification of the suitable elements of the SiO_2_ thin film obtained with an optimized ALD regime on a TiO_2_/Si substrate and a Si substrate

Name	At%	At% (depth profiling)
**SiO_2_/TiO_2_/Si**		
Si 2p	16.4	—
O 1s	58.8	63.4
N 1s	1.4	—
Cl 2p	1.1	3.8
C 1s	14.6	—
Ti 2p	7.7	32.8

**SiO_2_/Si**		
Si 2p	25.9	34.4
O 1s	60.5	62.9
N 1s	1.6	2.3
Cl 2p	1.0	0.5
C 1s	11.1	<1

**Fig. 8:**
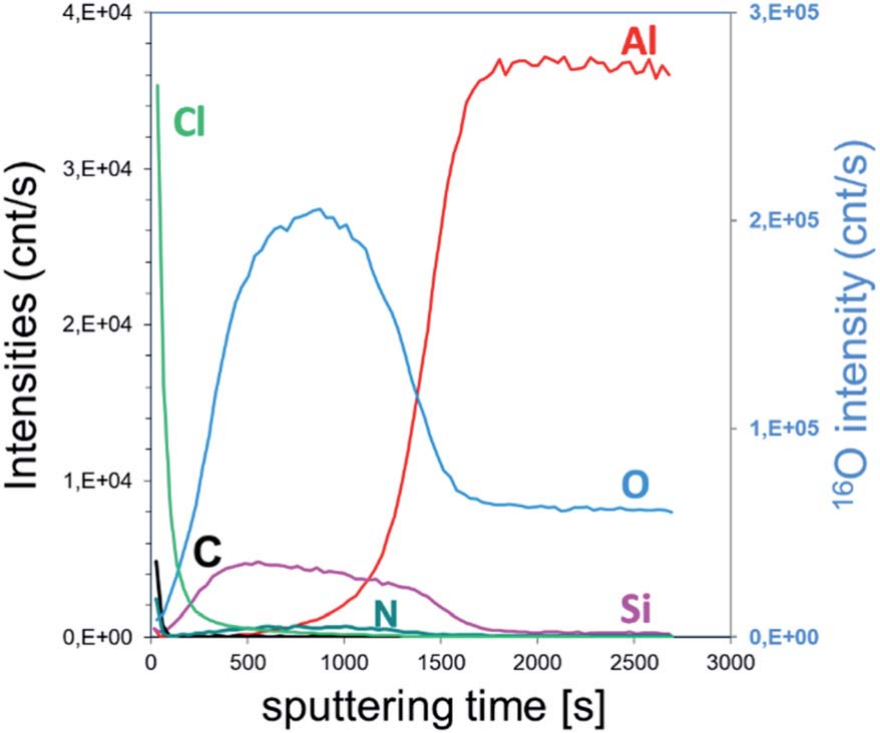
SIMS depth profile of porous SiO_2_ film obtained with 100 ms pulse of SiCl_4_, 2s of NH_3_ and H_2_O precursors.

The SIMS depth profiling of the synthesized SiO_2_ thick film exhibits a concentration of chlorine that rapidly decreases as a function of sputtering time (Fig. 8). Compared to the previous process, the intensity of Cl is starting a decade less, around 3.5 × 10^4^ cnts per s. Moreover, the amount of C and N is very low, which confirms the low level of NH_4_Cl contamination in a thick volume of SiO_2_.

**Fig. 9:**
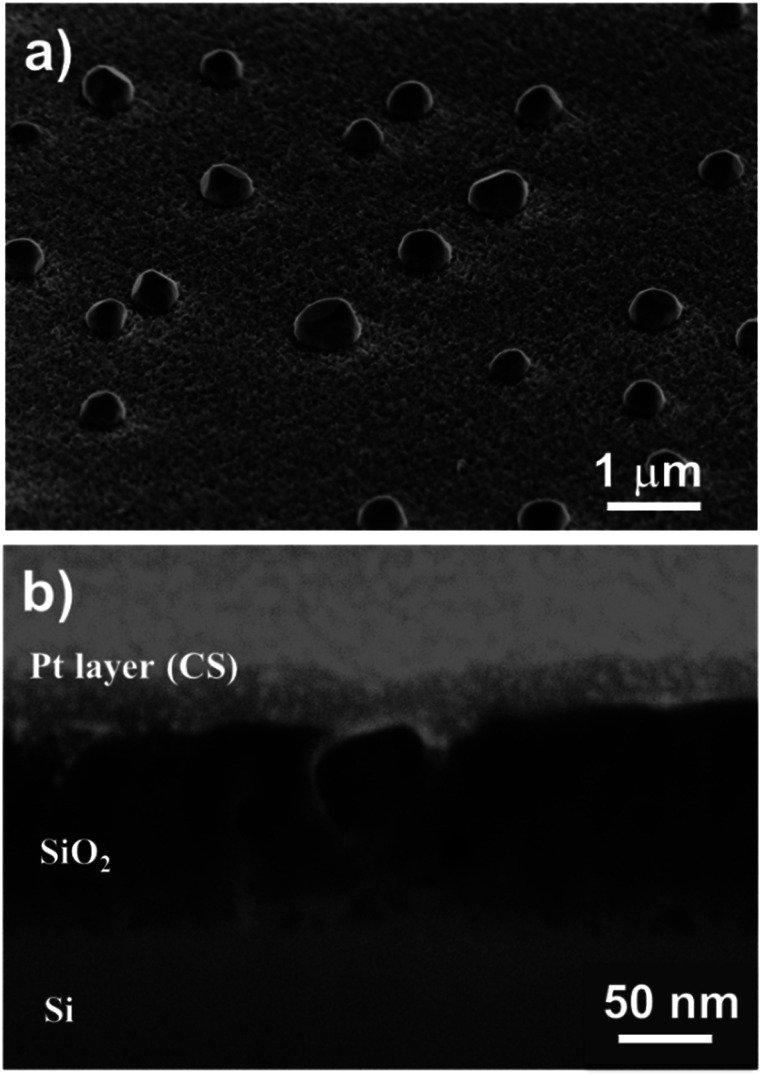
SEM images of the ∼260 nm thick ALD SiO_2_ (300 cycles) film at different magnifications: (a) 45° tilted view of the entire oxide film; (b) FIB cross-section that confirms the porous state of the film.

Furthermore, the higher concentration of chlorine close to the surface of the film indicated the slow dissociative chemisorption of water, which induced the desorption of HCl. This recombination clearly affected the growth mechanism of SiO_2_. As shown in Fig. 9, SEM analyses highlighted the porous state of the oxide film. In addition to the 200–500 nm diameter aggregates on the surface of the layer, the top and tilted view (Fig. 9a) revealed a SiO_2_ sponge-like structure. The porosity of the film was confirmed by SAXS where a periodical arrangement of pores could be fitted with an average radius of 130 Å (std dev 30% and most frequent radius ∼ 110 Å). The applied FIB cross-section (Fig. 9b) reveals the presence of 20–50 nm cavities (merging pores due to the preparation) and isolated pores of ∼15 nm. As explained by Puurunen in the ALD random deposition approach,^56^ if the growth per cycle is not constant, the increase in the surface roughness should be fast at the beginning of the growth and slow thereafter. This naturally indicates that a smaller number of ALD reaction cycles are required to fit a conformal deposition in a close-packed array as far as the growth rate is adjacent to an atomic monolayer.

**Fig. 10:**
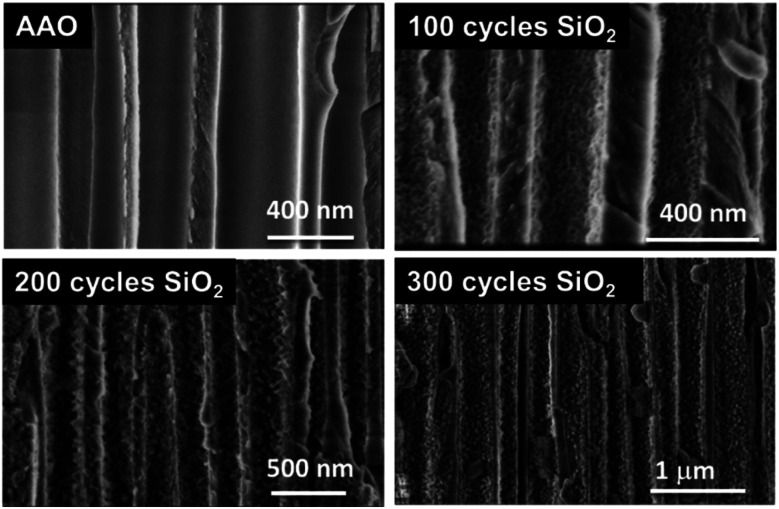
SEM images of ALD grown SiO_2_ films with various thicknesses (100–300 cycles) deposited in AAO membranes.

By considering the growth rate of ∼0.11 Å per cycle obtained in this process, it could explain why the SiO_2_ film is less “closed” as the one processed *via* the pure ALD approach. The sponge-like porous structure growth may be related to the limited surface diffusion of the by-products (NH_4_Cl, HCl) generated during each half cycle. Indeed, the diffusion/desorption of byproducts is slower in the case of low reaction temperature (here RT) and short purge time. In this case, residual water or byproducts (NH_4_Cl, HCl) are considered as surface fractions where the supplementary amount of the injected precursor will be adsorbed instead of the –Si–OH surface groups. This prevents the growth of SiO_2_ and leads to non-uniform porous thin films. Nevertheless, this peculiar structure is very attractive for applications that need to be processed at RT. Even if other techniques such as PE-ALD are able to produce SiO_2_ dense thin films with no impurities at reduced temperatures (50–80 °C) for specific applications,^41–43,57^ we evidenced our ability to fabricate a highly porous SiO_2_ layer at room temperature with a significant level of control of the contamination. This specific characteristic of this porous SiO_2_ layer is clearly transferred to complex and temperature-sensitive 3D materials. Hence, ALD SiO_2_ could be deposited in a porous anodic aluminum oxide (AAO) membrane, and the results are shown in Fig. 10. It clearly appears that the porosity obtained on planar substrates is perfectly transferable to 3D surfaces. This suggests that the ALD technique for SiO_2_ thin film synthesis could be applied to any 3D complex substrate. Further studies are in progress.

## Conclusions

Porous SiO_2_ thin films have been produced by ALD using a sequential exposure of SiCl_4_, NH_3_ and H_2_O at room temperature. The catalytic effect of ammonia has been exploited to optimize the saturation of the precursors and the extended purges. A relation between the significant porosity and the chemical saturation of the surface has been indicated using QCM, XPS, SIMS and SEM. It has also been demonstrated that this optimized process exhibited a decrease in the inherent inclusion of contaminants such as NH_4_Cl and HCl in the film. As demonstrated on AAO membranes, the transferability of this 2D process to 3D structures could extend the use of SiO_2_ films in several domains such as complex or high aspect ratio materials.

## Conflicts of interest

The authors disclose that there are there are no conflicts to declare.

## Supplementary Material

RA-010-D0RA01602K-s001
