# Supplementary material for: SiO2 thin film growth through a pure atomic layer deposition technique at room temperature
Source: RSC Adv. 2020 May 11;10(31):18073–81. doi: 10.1039/d0ra01602k (PMC9053598; doi:10.1039/d0ra01602k)
Supplement: RA-010-D0RA01602K-s001 [file RA-010-D0RA01602K-s001.pdf]

Electronic Supplementary Material (ESI) for RSC Advances.
This journal is © The Royal Society of Chemistry 2020

SiO_2 thin film growth through a pure Atomic Layer Deposition at Room-Temperature

Supporting information

S1:

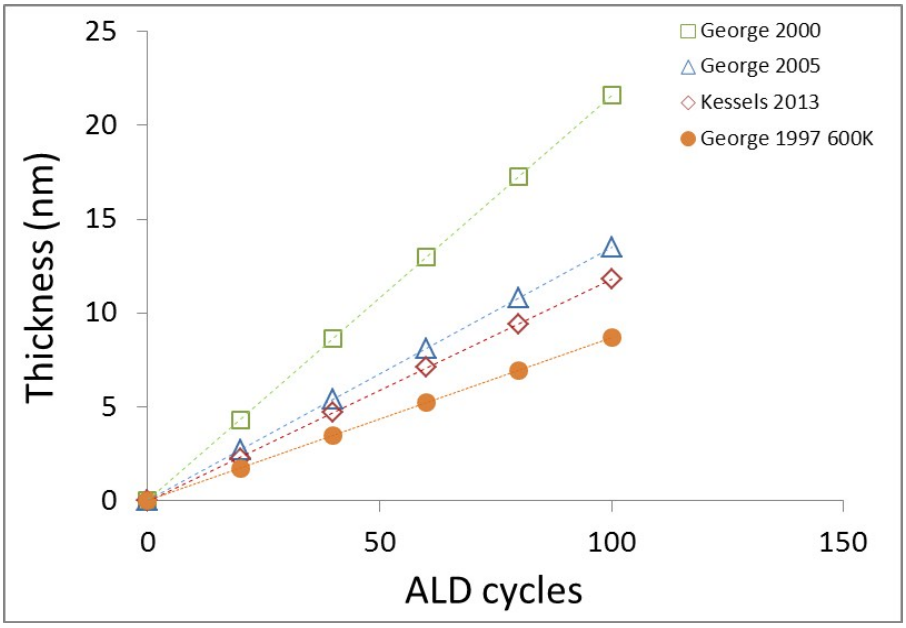

Evolution of SiO_2 film thickness as a function of ALD cycles based on the literature. All processes display a linear evolution with the number of cycles at room temperature [41,42,66] as well as high temperatures (600 K) [67].

S2:

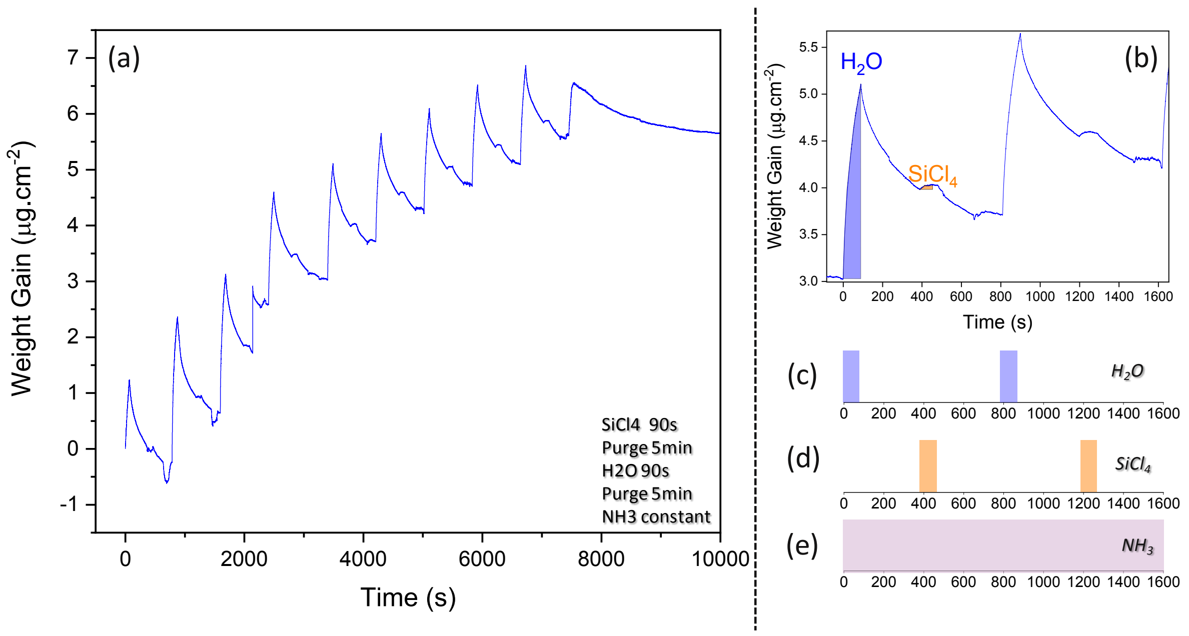

Growth kinetics of SiO_2 for sequential exposure of the surface to 90s of $SiCl_4$ and 90s of H_2O, indicating a growth rate of 0.6 µg.cm^{-2} per cycle. Panel (a) shows a typical zoom on 10 cycles of the total 2000 cycles deposited film. Panel (b) corresponds to the view of 2 cycles deposition. Lower panels represent the programmed exposures of H_2O (c), $SiCl_4$ (d) and NH_3 (e).

S3:

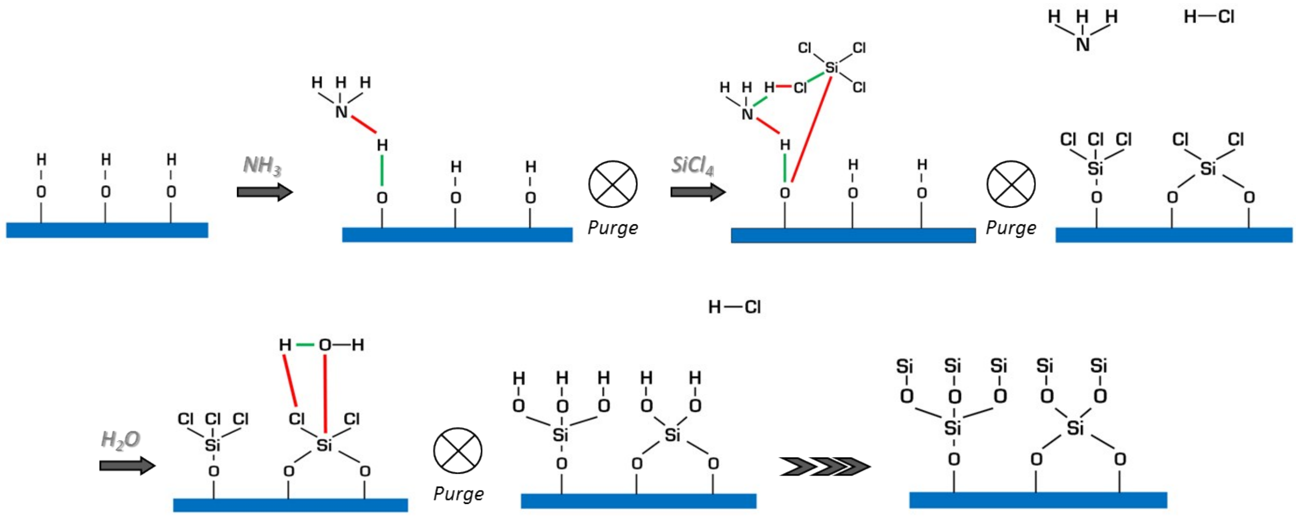

ALD room temperature growth of SiO_2 under NH_3 catalytic regime. Through the injection of NH_3, the O-H bond becomes weaker, -Si can easily react with –O at the surface to form –O-Si-$(Cl)_3$ or –O-Si(Cl_2)-O- ligands at the surface. -O coming from water injection directly react with –Si to finally form a SiO_2 monolayer.

S4:

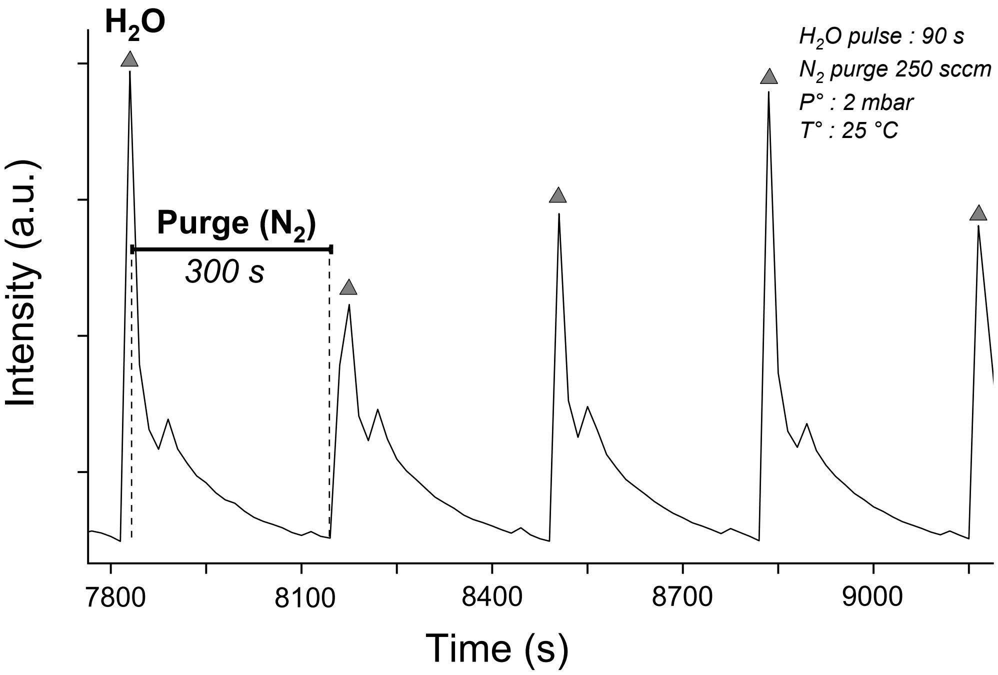

In-situ **RGA mass spectrometric monitoring of successive 90s H_2O pulses alternated with 300s N_2 purge. The panel corresponds to the measured intensity of H_2O (m/z = 18 uma).**

S5:

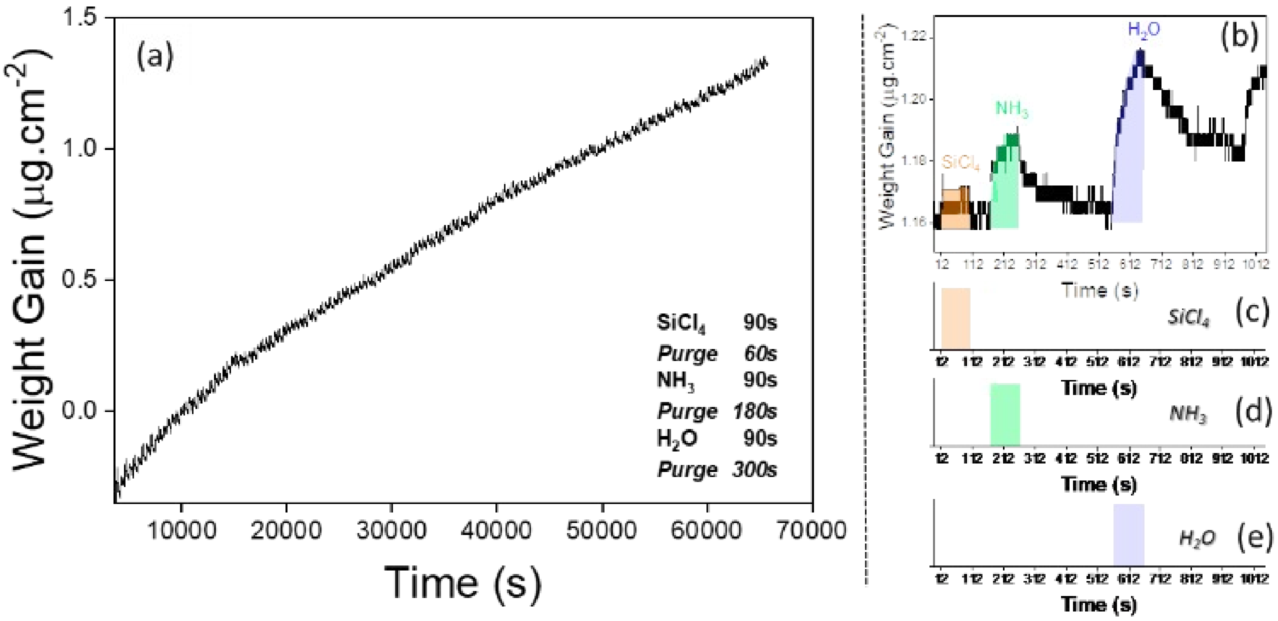

Fig. 5: Growth kinetics of pure ALD SiO_2 film with sequential exposure of the surface to 90s of $SiCl_4$, 90s of NH_3 and 90s of H_2O. (a) A typical zoom of the in situ monitoring of 500 loops deposition showing a growth rate of 0.02 µg.cm^{-2} per cycle. The panel (b) represents a zoom of the 60% advanced deposition. Lower panels represent the programmed exposures of $SiCl_4$ (c) NH_3 (d) and H_2O (e).

S6:

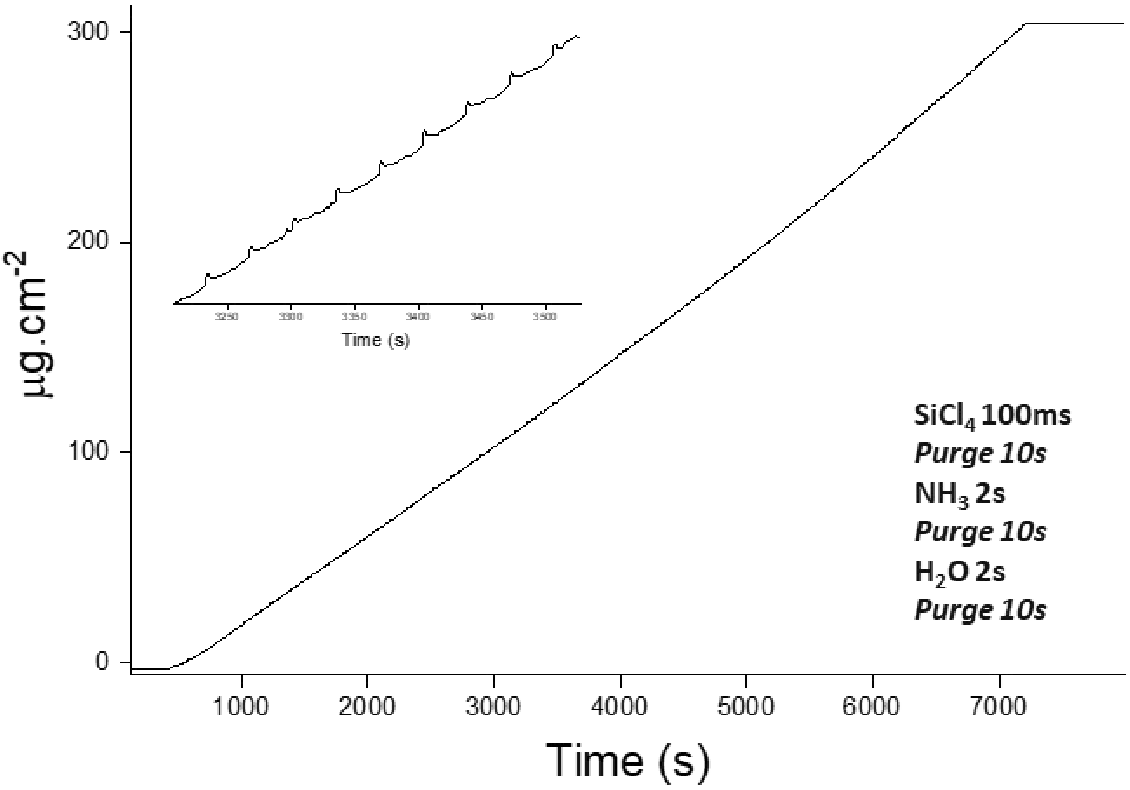

Growth kinetics of porous SiO_2 grown with sequential exposure of the surface to 100 ms of $SiCl_4$, 2s of NH_3 and 2s of H_2O. The in situ monitoring of 300 loops deposition corresponds to a growth rate of 1.5 µg.cm^{-2} per cycle. The insert represents a zoom of 10 cycles process.
